# Morphometric analysis for prioritizing critical areas of Urpash watershed in Sindh catchment of the lesser Himalayas using RS and GIS approach

**DOI:** 10.1371/journal.pone.0330503

**Published:** 2025-09-12

**Authors:** Mohmmad Idrees Attar, Sameena Naseer, Yogesh Pandey, Junaid Nazir Khan, Shabir Ahmad Bangroo, Mohd. Abul Hasan, Zubair Ahmad Khan, Afzal Husain Khan, Adil Majeed Tantray

**Affiliations:** 1 College of Agricultural Engineering and Technology, SKUAST, Srinagar, Kashmir; 2 Division of Soil Science, SKUAST, Srinagar, Kashmir; 3 Civil Engineering Department, College of Engineering, King Khalid University, Abha, Kingdom of Saudi Arabia; 4 Civil and Architectural Engineering Department, College of Engineering and Computer Sciences, Jazan University, Jazan, Saudi Arabia; Escola de Engenharia de São Carlos da Universidade de São Paulo: Universidade de Sao Paulo Escola de Engenharia de Sao Carlos, BRAZIL

## Abstract

Effective soil and water conservation is critical in fragile watersheds prone to erosion. However, assessing erosion susceptibility in ungauged watersheds remains challenging due to the lack of observed hydrological data This study addresses this gap by employing a comprehensive morphometric analysis integrated with remote sensing (RS) and Geographic Information Systems (GIS) to prioritize erosion-prone sub-watersheds within the ecologically sensitive and data-scarce Urpash watershed. Shuttle Radar Topography Mission (SRTM) Digital Elevation Model (DEM) data and ArcGIS 10.7 were used to analyze a 21.37 km² area. Key morphometric parameters—including linear, areal, and relief aspects—were assessed to understand watershed hydrology and erosion susceptibility. A total of 32 streams were identified, categorized into 1st to 3rd orders. Watershed shape indices, such as elongation ratio (R_e _= 0.65), form factor (R_f _= 0.33) and circularity ratio (R_c_ = 0.295), indicate an elongated shape, indicative of reduced surface runoff and erosion potential, along with higher sub-soil permeability. However, drainage parameters like drainage density (D_d _= 1.67 km/km^2^), stream frequency (F_s _= 1.49 km^-2^) and drainage intensity (D_i _= 0.89 km^-1^) pointed to the watershed’s susceptibility to flooding, gully erosion, and landslides due to slow surface runoff dispersion. Relief parameters such as basin relief (H = 1742.87 m), relief ratio (R_h _= 0.22) and ruggedness number (R_n_ = 2.9) reflect the watershed’s high relief and steep terrain, indicating a greater susceptibility to erosion. Using a compound parameter approach, the sub-watershed prioritisation ranked WS_3_ as the highest priority, followed by WS_4_, WS_5_, WS_6_, WS_2_, and WS_1_. By using RS and GIS-based morphometric analysis in an ungauged Urpash watershed, this study provides a geospatial framework for targeted soil and water conservation strategies, contributing to more precise watershed management in data-scarce and erosion-vulnerable regions.

## Introduction

Watershed prioritization has become a crucial focus in hydrology and natural resource management, particularly in regions vulnerable to soil erosion and flooding [1,2]. Morphometric analysis, the quantitative assessment of a watershed’s physical characteristics [3], has emerged as an essential tool for understanding the geomorphology and hydrological behaviour of river basins [4,5]. By examining aspects such as size, shape, and relief, this approach helps identify erosion-prone areas and guide soil and water conservation efforts [6,7]. Due to the watershed’s significance as the fundamental unit in hydrology [8,9] and its ability to show the interaction of soil and water resources, morphometry at the basin level is preferred, rather than conducting it on isolated channels [10]. This preference arises from the fact that a watershed represents an area where the primary runoff is directed towards a singular outlet [11,12] and acts as a vital unit in hydrology and plays a crucial part in determining the Earth’s landscape and governing the flow of water across various terrains [10]. Morphometric features of a catchment encompass its measurable and physical attributes, representing inherent features [13]. Additionally, they play a pivotal role in prioritizing erosion-prone zones for optimal soil and water management [14]. The study of morphometric analysis within watersheds has garnered significant attention from researchers and environmentalists due to its potential to unravel the intricate relationships between landforms, hydrological processes, and ecological systems [15]. By quantitatively assessing various aspects of a watershed, morphometric analysis offers valuable insights into the basin’s geomorphological characteristics, making it a powerful tool for understanding the dynamic interactions that shape landscapes and influence environmental processes [16].

Research on morphometry has progressed significantly, utilizing both conventional [17–19] and advanced techniques such as Remote Sensing (RS) and Geographic Information Systems (GIS) [4,20–22]. However, RS and GIS techniques allow for more effective evaluation of drainage basin changes and offer greater flexibility in spatial information analysis [23]. Image interpretation techniques of RS save time compared to ground surveys and when combined with field investigations, produce valuable outcomes [24]. The process of prioritizing watersheds holds paramount importance within natural resource management systems [25]. Various researchers have undertaken morphometric analyses to prioritize critical subwatersheds in different regions worldwide. For instance, Balasubramanian et al. [26] focused on the lower Bhavani basin in Tamil Nadu, India; Nwilo et al. [27] conducted their study in the Imo River Basin; Nasir et al. [28] examined subwatersheds of the Swat River; Inyele et al. [29] investigated the Thiririka Watershed in Kenya; Shekar et al. [30] analyzed the Peddavagu River Basin in India and Topno et al. [31] studied the Rarhu watershed. These studies utilized morphometric analysis techniques to identify and prioritize areas vulnerable to soil erosion, contributing to the understanding and management of erosion risks in diverse geographical contexts. Additionally, land use changes significantly influence the morphometry of a watershed by altering drainage characteristics, runoff patterns, and erosion susceptibility. Several studies have explored these interactions using remote sensing and GIS techniques. Worachairungreung et al. [32] examined agricultural land loss by analyzing changes in land use and land cover, while Hassan et al. [33] assessed the effect of climate change on wetland areas in West Iraq using satellite data and GIS techniques. Furthermore, Rattanarat et al. [34] investigated how government policies influence land use and land cover changes over a 30-year period.

Despite significant advancements in watershed prioritization research, studies specifically addressing the ecologically fragile and ungauged Urpash watershed remain scarce. This region faces increasing environmental pressures, highlighting the urgent need to prioritize sub-watersheds for targeted conservation efforts. The lack of comprehensive research in the Urpash watershed further underscores the necessity for a detailed analysis. This study goes beyond conventional morphometric analysis by integrating erosion risk assessment through the Revised Universal Soil Loss Equation (RUSLE) model, thereby validating the prioritization derived from morphometric parameters. By employing both the compound parameter method [17] and RUSLE [35,36], the research ensures a more robust evaluation of sub-watershed vulnerability to erosion, enhancing the reliability of prioritization results. This novel approach not only bridges the knowledge gap in watershed prioritization for the Urpash watershed but also establishes a scientifically validated framework that can be applied to other ecologically sensitive and understudied regions. The findings aim to contribute to sustainable natural resource management and inform future conservation strategies.

## Study area

**Fig 1:**
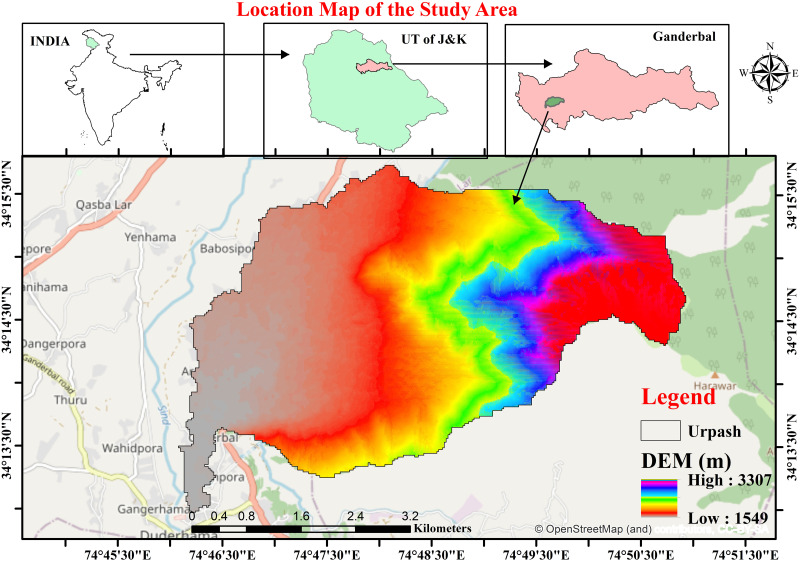
Location map of Urpash watershed (Source: https://earthexplorer.usgs.gov/).

The study focuses on the assessment of various morphometric features of the Urpash Watershed, located in the district of Ganderbal, J&K, India, covering an area of approximately 21.37 km². Fig 1 displays the location map of the basin. The region’s topography is characterized by diverse landforms, ranging from rugged mountainous terrain in the northeastern regions to gently rolling hills and plains in the southwestern. Mountain, grainy, karewa and soils rich in organic matter are the primary soil types present in the watershed and the predominant soil texture class found is clayey loam [36,37]. The watershed has 124 wet days on average every year, with an average rainfall of 1242 mm [36]. Regarding site access and permits, no specific permissions were required for conducting field checks in this study. The selection of the Urpash watershed was based on its fragile nature and susceptibility to erosion, making it a suitable candidate for morphometric and hydrological assessments. Since the study did not involve restricted or protected areas, nor did it require any interventions affecting the local environment, obtaining formal permits was not necessary.

**Fig 2:**
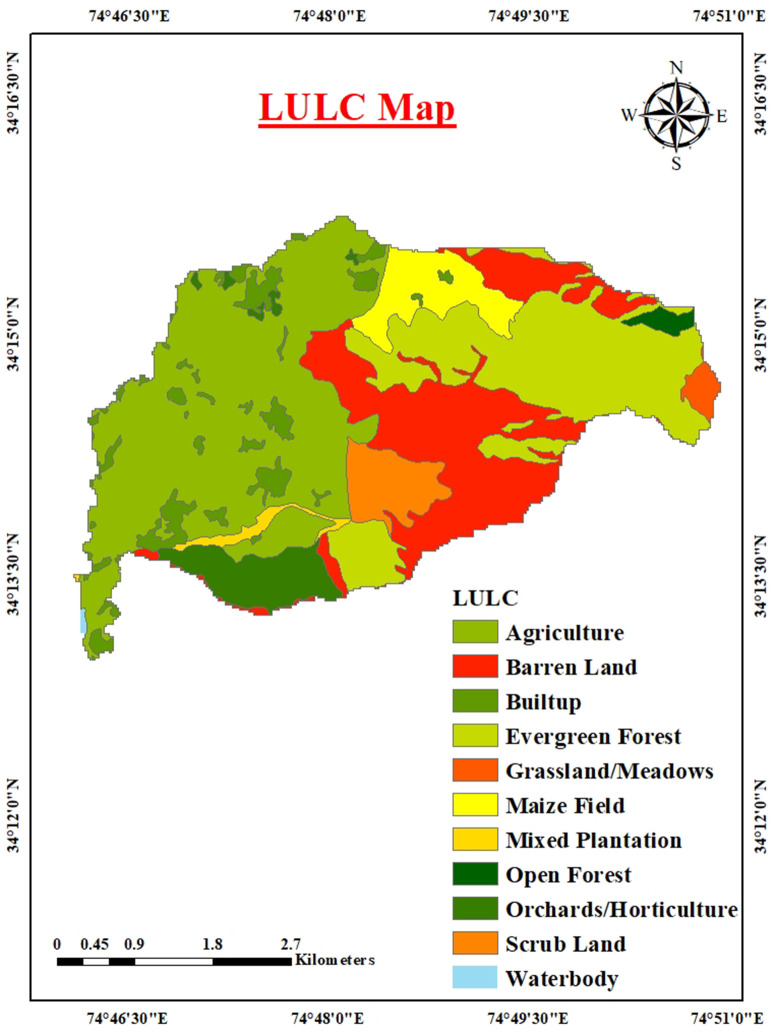
Land Use/Land Cover (LULC) classification map of the Urpash watershed (Source: generated from data obtained from https://earthexplorer.usgs.gov/).

The Urpash Watershed, entirely rural in nature, supports a diverse landscape dominated by agricultural land use, covering 7.81 km² (47.84%) of the total area, followed by evergreen forest, which occupies 4.74 km² (32.96%). Other significant land uses include barren land at 4.24 km² (38.32%), built-up areas covering 1.11 km² (32.27%), and orchards/horticulture, which span 1.07 km² (9.42%). Additional land use categories include maize fields at 1.06 km² (7.82%), mixed plantations at 0.20 km² (5.80%), scrubland at 0.81 km² (4.96%), and grassland/meadows at 0.19 km² (2.56%). Open forest and water bodies account for 0.15 km² (2.17%) and 0.02 km² (0.74%), respectively (Fig 2). Agriculture and horticulture, particularly apple and walnut production, along with crops like paddy, maize, and mustard, are central to the local economy.

Hydrologically, the Urpash Watershed is part of the Sindh River catchment, with the Urpash Canal (locally known as the Bijli Canal) serving as the principal watercourse traversing the area. The canal sustains diverse aquatic and terrestrial ecosystems within its riparian zones and is important in supplying water for residential and agricultural purposes. While no direct river discharge data is available for the canal, the region’s high average rainfall and the watershed’s small area contribute to its fast hydrological response, meaning rainwater reaches the main channel quickly due to limited travel distance [37]. Additionally, the watershed is enriched by several smaller streams and tributaries, contributing to its extensive hydrological network. Water demand in the Urpash Watershed is increasing due to agricultural expansion and population growth, leading to seasonal water shortages. Land use changes and climate variability further stress the ecosystem [32,34,38]. A geomorphological study is essential to assess the watershed’s characteristics and guide sustainable soil and water conservation efforts.

## Materials and methods

**Fig 3:**
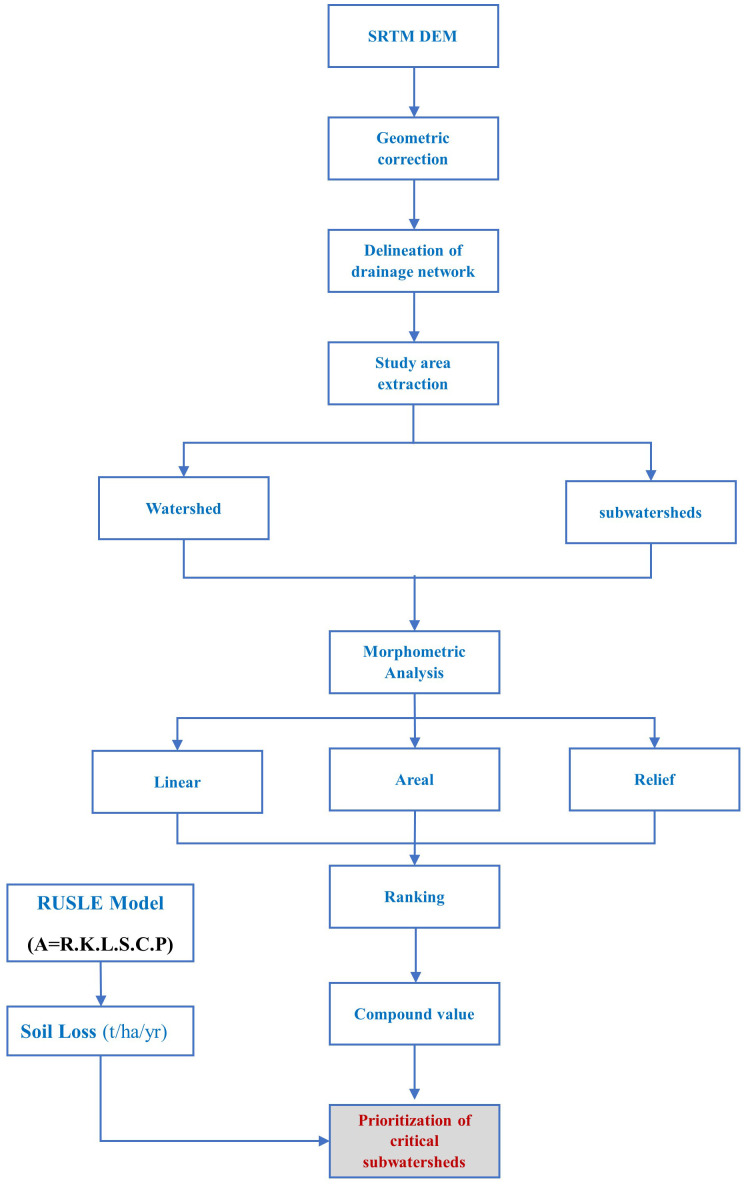
Flowchart of the methodology adopted in the study.

**Fig 4:**
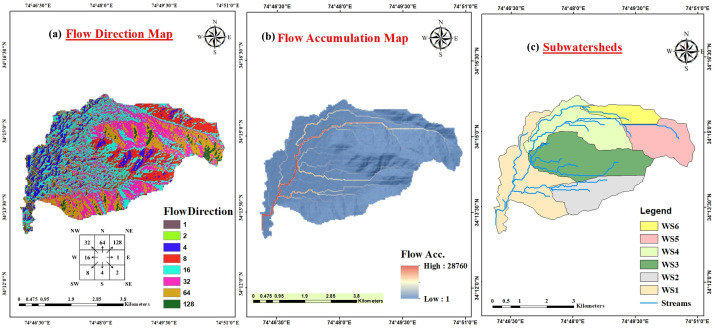
Hydrological characteristics of the watershed. (a) Flow direction map and (b) Flow accumulation map of the Urpash watershed (c) Delineated subwatersheds of Urpash watershed (Source: generated from the data obtained from https://earthexplorer.usgs.gov/).

SRTM DEM of 30m resolution and ArcGIS were employed for assessing various morphometric parameters. The choice of a 30m DEM is appropriate for a watershed of 21.37 km², as it provides a balanced resolution for capturing the necessary topographic details without overwhelming computational requirements. The DEM was further validated using SOI toposheets. This process involved aligning the DEM with the topographic features represented in the toposheets to correct any discrepancies and ensure that the digital elevation model accurately reflects the terrain. Additionally, any sinks within the DEM were identified and filled to prevent errors in hydrological analysis. The methodology adopted to evaluate various parameters is presented in Fig 3. The morphometric analysis starts with the creation of flow direction and flow accumulation maps (Fig 4 a, b) in ArcMap which are later used to obtain other parameters. By establishing a pour point for each sub-basin, the boundaries of the 6 subwatersheds were derived which are shown in Fig 4c. The pour point represents the specific spot in the basin where water from the entire basin converges and joins the primary stream.

In this study, several morphometric features that have been evaluated are presented in Table 1 along with their respective formulae and references. The selection of these parameters was guided by their proven relevance in influencing watershed hydrology, runoff characteristics, and soil erosion. Multicollinearity among the selected morphometric parameters was checked using correlation analysis to ensure that highly correlated parameters did not skew the results. Additionally, alternative parameter sets were tested to evaluate their impact on the outcomes, and the findings remained consistent, reinforcing the reliability of the chosen parameters. Finally, the identification of the priority of sub-basins was conducted using a compound parameter approach and RUSLE (Revised Universal Soil Loss Equation) model [36]. The reason for defining watershed prioritization classes is to identify critical areas most vulnerable to erosion, allowing for targeted conservation efforts. The rationale for choosing the compound parameter approach over other widely used prioritization techniques (e.g., Weighted Overlay Analysis, AHP, Fuzzy Logic, or MCDM) is grounded in its simplicity, transparency, and established application in morphometric studies [39,40]. In this study, the relevant linear, relief and areal parameters were selected to assess the critical areas of the Urpash watershed since these parameters influence the surface runoff and soil loss occurring in the watershed [41,42]. Linear and relief features have a direct influence on soil loss, with higher values indicating more erosion in a basin [43–45]. Conversely, areal/shape parameters demonstrate an inverse relationship with soil loss, with lower values signifying more erosion [46]. Accordingly, sub-watersheds with the highest values in linear and relief parameters are ranked first, followed by those with successively lower values. Conversely, sub-watersheds with the lowest values in areal/shape parameters are ranked first, with higher values receiving lower ranks [47,48]_._ Prioritisation using the RUSLE model was employed to evaluate the priority of sub-watersheds of Urpash based on soil loss values. These data sources, combined with limited field checks, have enabled us to capture crucial morphometric features, such as channel length, basin area, relief parameters, and drainage patterns. Understanding these features is vital for comprehending the geomorphological processes that influence the formation and dynamics of the watershed [49]. It is anticipated that the study’s findings will significantly contribute to the scientific understanding of watershed dynamics and guide policymakers and environmentalists in developing effective strategies for sustainable watershed management and conservation.

**Table 1:** Morphometric parameters used in the study, their respective formulae and the references.

S. No	Morphometric parameters	Formula	Reference
A	Drainage network		
1	Stream order	Hierarchical Rank	Strahler’s method [50]
2	Total stream order	Sum of stream order	
3	Stream number (Nu)	Nu = N1 + N2 + … + Nn	Horton’s method [18]
4	Stream length (Lu) (km)	Length of the stream	Strahler’s method [51]
5	Stream length ratio (Lur)	$\text{Lur}=\frac{L_{u}}{L_{u}-1}$	Strahler’s method [51]
6	Bifurcation ratio (Rb)	$\text{Rb}=\frac{N_{u}}{N_{u}+1}$	Schumm’s method [52]
B	Basin geometry		
7	Basin perimeter (P)	GIS software analysis	
8	Basin Length (Lb) (km)	GIS software analysis	
9	Basin area (A) (km2)	GIS software analysis	
10	Form factor ratio (Rf)	$\text{Rf}=\frac{A}{L_{b}^{2}}$	Horton’s method [53]
11	Elongation ratio (Re)	Re = √A/pi/Lb	Schumm’s method [52]
12	Shape factor ratio (Sf)	$\text{Sf}=\frac{L_{b}^{2}}{A}$	Horton’s method [18]
13	Circularity ratio (Rc)	$\text{Rc}=\frac{A}{P}$	Strahler’s method [51]
14	Relative perimeter (Pr) (km)	Pr = A/P	Schumm’s method [52]
C	Drainage texture analysis		
15	Drainage density (Dd) (km/km2)	$\text{Dd}=\frac{L_{u}}{A}$	Horton’s method [53]
16	Stream frequency (Fs)	$\text{Fs}=\frac{N_{u}}{A}$	Horton method [53]
17	Drainage intensity (Di)	$\text{Di}=\frac{F_{s}}{D_{d}}$	Faniran’s method [54]
18	Length of overland flow (Lo) (km)	Lo = 1/(Dd × 2)	Horton’s method [18]
19	Drainage texture (Dt)	Dt = Nu/P Where Nu = No. of streams in a given order and P = Basin perimeter	Horton’s method [18]
20	Infiltration number (If)	If=Dd × Fs	Zavoiance’s method [55]
D	Relief characterization		
21	Maximum basin height (Z) (m)	GIS software Analysis	
22	Minimum basin height (z) (m)	GIS software Analysis	
23	Total basin relief (H) (m)	H = Z-z	Strahler’s method [50]
24	Relief ratio (Rh)	Rh = H/Lb	Schumm’s method [52]
25	Relative relief ratio (Rhp)	Rhp=(H × 100)/P	Melton’s method [55]
26	Ruggedness number (Rn)	Rn = Dd×(H/1000)	Patton and Baker’s method [56]

## Results and discussion

### Quantification of linear features

The linear morphometric characteristics are calculated in Table 2.

**Table 2:** Linear morphometric parameters of Urpash watershed.

Stream order (u)	Stream number (N_u_)	Total stream numbers	Stream length (L_u_) (km)	Total stream length (km)	Mean stream length (L_sm_) (km)	Cumulative mean stream length (L_sm_) (km)	Stream length ratio (L_ur_)	Bifurcation ratio (R_b_)	Mean bifurcation ratio (R_bm_)	Basin length (km)
**1**	17	32	20.80	35.80	1.22	1.22				
**2**	10		10.75		1.07	2.29	0.87	1.7	1.85	8
**3**	5		4.25		0.85	3.14	0.79	2		

#### Stream order (u).

**Fig 5:**
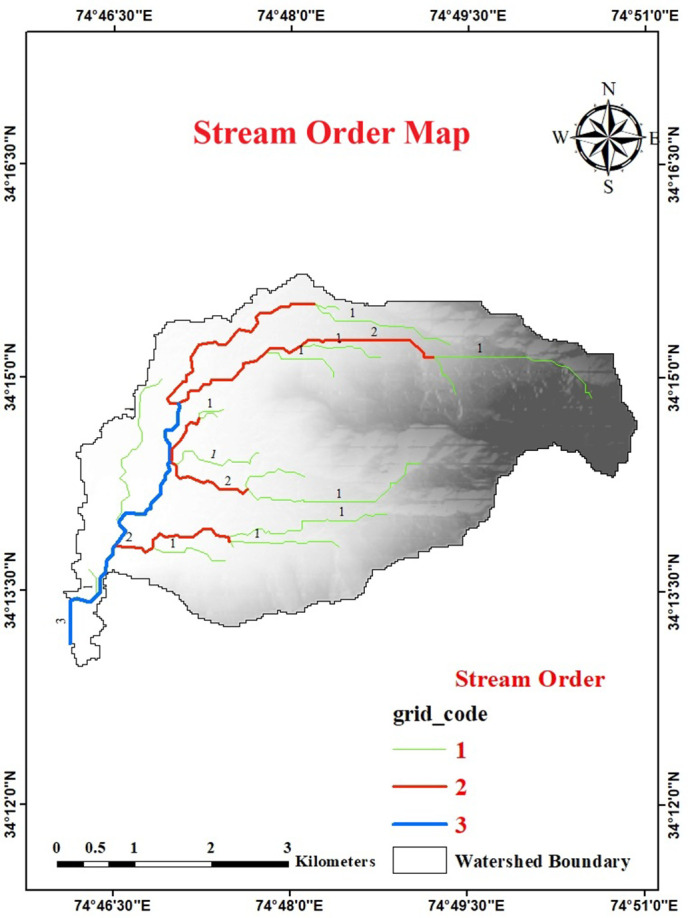
Stream order map of the Urpash watershed, depicting the hierarchical arrangement of streams based on Strahler’s classification system. (Source: generated from the data obtained from https://earthexplorer.usgs.gov/).

The initial step in the examination of the drainage basin is to designate stream orders [15,57]. Stream order is the ordinal designation given to a segment of a stream within a river network, determined by its placement in the hierarchical branching configuration of the drainage system [58]. Using Strahler’s method [59], the Urpash watershed is identified as a 3rd order basin exhibiting a dendritic drainage pattern, where higher stream orders signify greater discharge (Fig 5). This dendritic configuration indicates uniformity in the geological substrate and the absence of structural control [60,61].

#### Stream number (N_u_).

Stream number denotes the number of stream segments that exist in each order [62]. The results revealed that the Urpash watershed had 32 streams linked with all 3 orders covering an area of 21.37 km^2^. The majority of the total stream count (17) is comprised of 1^st^ order streams while 2^nd^ order constitutes 10 streams and 3^rd^ order constitutes five streams. Hence, the principle that a lower stream order corresponds to a greater number of streams is consistently observed across the entire watershed [18,63]. A higher stream number is associated with lesser permeability and infiltration and vice-versa [20]. Moreover, watersheds with a higher number of first-order streams are more prone to flash flooding during intense rainfall events compared to those with fewer first-order streams [64].

#### Stream length (L_u_).

**Fig 6:**
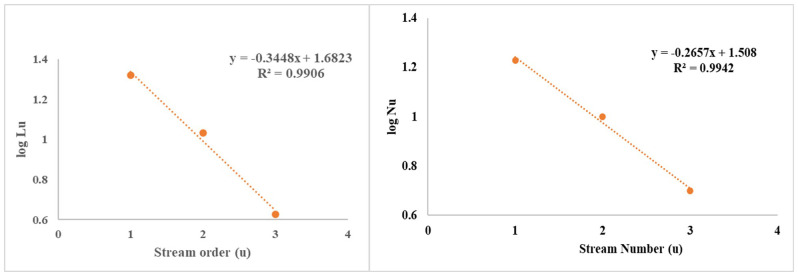
Graphical representation of the relationship between stream order, stream number, and stream length in the Urpash watershed.

Stream length represents the average length of streams within various orders present in a drainage basin [65]. The overall stream length of the Urpash watershed was found to be 35.80 km, in which the 1^st^ order stream length was 20.80 km, the 2^nd^ order length was 10.75 km and the 3^rd^ order stream length was found to be 4.25 km. The study has shown that results adhere to Horton’s Law of L_u_ and N_u_ [18] (Fig 6), indicating that the shorter lengths of higher-order streams compared to lower-order streams suggest a steeper basin profile, which contributes to faster runoff within the watershed [66,67].

#### Mean stream length (L_sm_).

L_sm_, a dimension-based attribute, reveals the dimensions of elements within a drainage channel and the corresponding contributing areas [51]. In the Urpash watershed, the L_sm_ for first, second and third-order streams was calculated as 1.22, 1.07 and 0.85 respectively.

#### Stream length ratio (L_ur_).

The ratio of L_sm_ of a given order to the next higher order is defined as the stream length ratio [6]. L_ur_ values of the Urpash watershed varied from 0.79–0.87, indicating that the watershed is in an early stage of geomorphic development [68].

#### Bifurcation ratio (R_b_).

R_b_ is associated with the branching arrangement of the streams of the watershed. According to Schumm [52], it is calculated by dividing the sum of streams (N_u_) of any order by the streams of the next higher order (N_u_ + 1). The R_b_ of the Urpash watershed varies from 1.7 to 2, with a mean bifurcation ratio of 1.85 which suggests less structural disturbance [69]. The slightly higher bifurcation ratio indicates the high overland flow and thus high erosion risk. In addition, high R_b_ values suggest an elongated shape, whereas lower values suggest a more circular or rounded shape [70,71]. This indicates that WS_4_, WS_5_, and WS_6_ experience relatively higher levels of disturbance compared to other sub-watersheds, while WS_1_ and WS_2_ are relatively more sustainable. Therefore, based on the R_b_ the Urpash watershed can be considered as a watershed which has a low to medium runoff generation potential.

#### Basin length (L_b_).

L_b_ represents the greatest extent of the basin or displacement between stream confluence and the farthest point on the watershed boundary [72,73]. Employing ArcGIS 10.7, the computed basin length in our study area was found to be 8 km. Subsequently, the analysis revealed that within the subwatersheds, WS_3_ exhibited the highest L_b_ value at 4.54 km, while WS_5_ showcased the lowest value, measuring 2.92 km, suggesting that runoff water in WS_5_ would travel longer than other subwatersheds. These findings underscore the significant variability in basin length across different subwatersheds, highlighting potential implications for hydrological processes and land management strategies within the study area.

### Quantification of areal parameters

The estimated areal morphometric features of Urpash are presented in Tables 3 and 4 respectively.

**Table 3:** Basin parameters of Urpash watershed.

Basin perimeter (km)	Basin area (km^2^)	Form factor (R_f_)	Elongation ratio (R_e_)	Shape factor ratio (S_f_)	Circularity ratio (R_c_)	Relative perimeter (P_r_)
30.16	21.37	0.33	0.65	2.995	0.295	0.71

**Table 4:** Drainage and relief parameters of Urpash watershed.

Drainage Density (km/km^2^)	Stream frequency (F_s_) (km^-2^)	Drainage intensity (D_i_)	Infiltration No. (I_f_)	Length of overland flow (km)	Drainage texture (D_t_)	Max. basin height (Z) (m)	Min. basin height (z) (m)	Basin relief (H) (m)	Relief ratio (R_h_)	Relative relief ratio (R_hp_)	Ruggedness No. (R_n_)
1.67	1.49	0.89	2.48	0.29	1.06	3295	1552.13	1742.87	0.22	5.77	2.90

#### Basin area (A).

Watershed area (A) denotes the entirety of the land surface within a watershed projected onto a horizontal plane. The calculated basin area of the Urpash watershed using ArcGIS 10.7 software was obtained as 21.37 km^2^. Based on the area, WS_3_ had the largest area (5.18 km^2^) and WS_6_ was the smallest one (1.38 km^2^). The area of the basin stands as a paramount watershed attribute in drainage study as it directly correlates with the volume of water contained within the watershed [74,75]. Considering the relationship outlined between watershed area and other hydrological factors, larger watersheds such as WS_1_, WS_3_, and WS_4_ are expected to exhibit higher mean discharge and mean annual runoff [76], while smaller watersheds like WS_2_, WS_5_, and WS_6_ are anticipated to demonstrate lower values of these parameters.

#### Basin perimeter (P).

The perimeter defines the outer border of the watershed, encompassing its total area. It serves as an indicator of the watershed’s size and shape. The perimeter of the Urpash watershed using ArcGIS 10.7 was found to be 30.16 km. Subsequently, within the subwatersheds, WS_1_ exhibited the highest P value, while WS_6_ showcased the lowest value.

#### Elongation ratio (R_e_).

R_e_ is the ratio of the diameter of a circle having the same areal extent as a study area to L_b_ of the study area [52,77]. The value of the R_e_ in the Urpash was calculated as 0.65, which indicates the elongated shape of the Urpash watershed [67,78]. WS_6_ had the lowest R_e_ value indicating its vulnerability while the remaining five subwatersheds exhibited similar R_e_ values.

#### Circularity ratio (R_c_).

Re represents the ratio of the watershed’s area to the area of a circle with a perimeter equal to that of the watershed [79,3]. It is influenced by factors such as stream length, geological formations, LULC, climatic conditions, stream frequency, topography, and watershed gradient. The value of R_c_ was found to be 0.295, which means Urpash is an elongated-shaped basin [80], implying low peaked runoff and reduced erosion potential [81]. Among the subwatersheds, WS_1_, WS_4_ and WS_6_ showed lower values of R_c_, while the remaining subwatersheds exhibited higher values.

#### Form factor (R_f_) and shape factor (S_f_).

It is defined as the ratio of basin area (A) to basin length (L_b_) [53]. The low R_f_ value of 0.33 indicates that the elongated shape of the Urpash watershed [51] results in lower runoff, a longer concentration time, and reduced susceptibility to flooding and erosion [82]. The Shape factor (S_f_) which is defined as the reciprocal of R_f_ is also used to evaluate basin shapes, and its value was 2.995. The R_f_ values with the watershed are higher in WS_5_ and lower in the rest of the subwatersheds.

#### Drainage density (D_d_).

**Fig 7:**
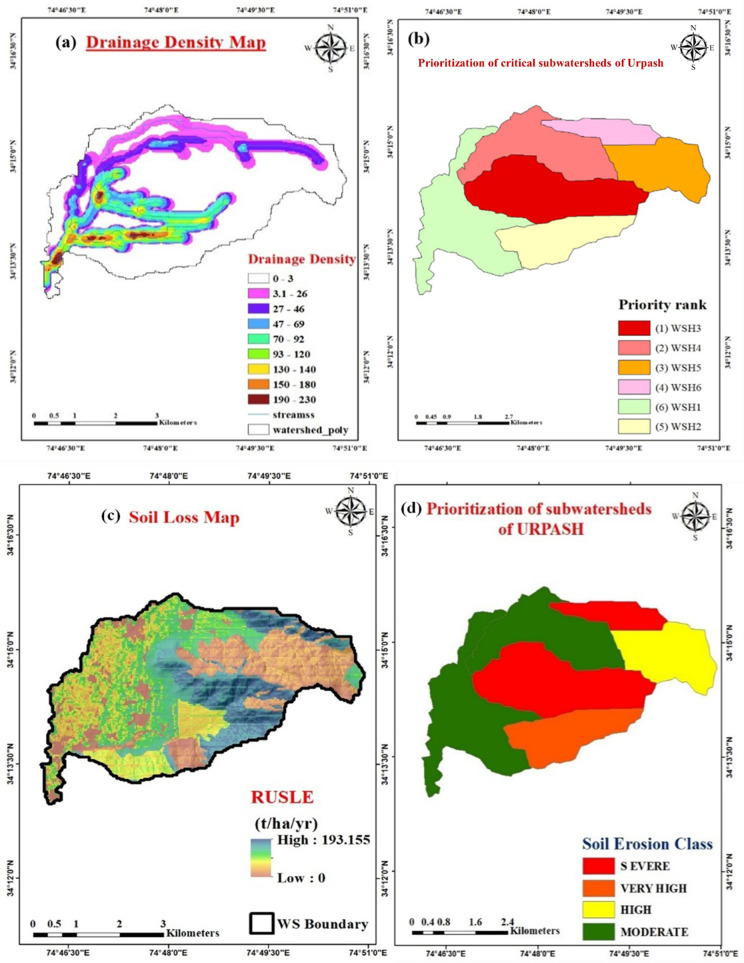
Morphometric and erosion-based prioritization. (a) Drainage density map of Urpash watershed (b) Priority map of subwatersheds in the Urpash watershed based on compound parameter values derived from morphometric analysis, highlighting areas most susceptible to erosion (c) Erosion status map of the Urpash watershed, showing regions with different levels of soil erosion risk as identified through the RUSLE model (d) Priority map of subwatersheds of Urpash using RUSLE model (Source: generated from the data obtained from https://earthexplorer.usgs.gov/).

D_d_ represents the ratio of the combined length of all streams within a watershed to the area of that watershed (km/km^2^) [53,3,83]. It indicates the proximity of drainage lines [13,84]. The assessment of drainage density serves as a valuable quantitative measure of surface-water flow and sediment potential from the watershed [85–87]. The lower D_d_ value of 1.67 km/km^2^ suggests that the Urpash watershed is either situated on erosion-resistant surfaces or has a highly permeable substratum, reducing surface runoff [50]. It ranged from 1.09 in WS_5_ to 2.36 in WS_4_. The drainage density map is displayed in Fig 7a.

#### Stream frequency (F_s_).

It represents the total count of stream channels within a specified area [18,3]. F_s_ of the Urpash watershed was obtained as 1.49 km^-2^, which indicates rich vegetation cover. In this study, F_s_ ranged from 1.01 in WS_5_ to 2.17 in WS_6_. A direct correlation between F_s_ and D_d_ was observed [69]. Lower F_s_ and D_d_ values correspond to reduced runoff rates, consequently lower erosion risks.

#### Drainage intensity (D_i_).

It is defined as the ratio of F_s_ and D_d_ [54,3,83]. The calculated D_i_ value of Urpash was 0.89 km^-1^. The low values of D_d_, F_s_ and D_i_ indicate that watershed runoff can not immediately be taken out which is the cause of Urpash being more prone to hazards such as soil loss, swamping and landslips.

#### Infiltration number (I_f_).

It is the product of D_d_ and F_s_ and serves as an indicator of the infiltration characteristics of the basin [88,87,89]. The I_f_ of the Urpash watershed was estimated to be around 2.48 which indicates runoff will be low. The I_f_ values in the Urpash watershed ranged from 1.02 in WS_5_ and 4.10 in WS_4_.

#### Length of overland flow (L_o_).

L_o_ refers to the distance travelled by overland flow across the terrain before it gathers into main channels [18,86], exhibits an inverse relationship with the gradient of the channel and is significantly influenced by soil properties [74,90]. A higher L_o_ value signifies that water has to traverse a relatively longer path before joining the stream channels [91]. In the Urpash watershed, the L_o_ was obtained as 0.29 km indicating a lower distance to the main channel. The L_o_ value is highest at WS_5_ and lowest at WS_4_.

#### Drainage texture (D_t_).

D_t_ signifies the comparative arrangement of the stream network and is defined as the ratio of total streams and Perimeter [18,85]. It reflects the topography’s underlying lithology, infiltration capacity, and overall relief characteristics [92]. In the Urpash watershed, the drainage texture value was 1.06, classifying it within the coarse drainage texture group [18]. This implies favourable permeability, efficient infiltration rate, reduced runoff capacity, and notable groundwater recharge. The D_t_ values with the watershed are higher in WS_2_, WS_3_ and WS_4_ and lower in WS_1_, WS_5_, and WS_6_.

### Quantification of relief parameters

The calculated relief morphometric features are presented in Table 4.

#### Basin relief (H).

Basin relief (H) represents the greatest vertical span between the lowest point (outlet) and the highest elevation within the basin [93]. A larger vertical span implies a steeper basin. Basin relief directly influences runoff and sediment transport [94,95]. Consequently, watersheds such as WS3 and WS4, characterized by greater relief, are at a heightened risk of soil erosion. The basin relief of the Urpash watershed was 1742.87 m, which indicates the steeper nature of the Urpash watershed.

#### Relief ratio (R_h_).

R_h_ is the ratio of basin relief (H) to maximum basin length (L_b_) [44,52]. This ratio serves as an indicator of slope gradient and erosion intensity within a watershed. As the watershed area and its dimensions decrease, R_h_ value starts to increase [96]. The relief ratio serves as a gauge for the steepness of the terrain, erosion intensity, and the potential energy available for water and sediment movement [1,97]. In the Urpash watershed, the relief ratio was found to be 0.22, which indicates more relief and slope of the Urpash watershed. In this study, the higher and lower values of the R_h_ have been found at WS_5_ and WS_1_ respectively.

#### Relative relief ratio (R_hp_).

R_hp_ is the ratio of basin relief (H) to the basin perimeter [45,98]. The R_hp_ of the Urpash watershed was 5.77, which indicates more relief and steepness of the Urpash.

#### Ruggedness number (R_n_).

It is a measure of surface unevenness [3,99]. A quantitative metric known as the “ruggedness number” or “ruggedness index,” is used to evaluate the topographic complexity or roughness of a terrain or landscape. The R_n_ uses the gradient and span of slope to measure the instability of the basin [99]. The ruggedness number of the Urpash watershed was calculated as 2.9, which indicates that the study area has an uneven landscape and is highly prone to soil loss. In this study, the higher and lower values of the R_n_ have been found at WS_4_ and WS_1_ respectively, indicating that WS_1_ is more suitable for agricultural activities compared to the other subwatersheds [100].

### Prioritization of subwatersheds and recommendation

This study bridges this gap by integrating conventional morphometric analysis with soil erosion assessment using the Revised Universal Soil Loss Equation (RUSLE) model, ensuring a more comprehensive and scientifically validated prioritization approach. Table 5 presents a summary of the selected morphometric parameters of the six subwatersheds within the Urpash region. The prioritization of these subwatersheds was determined using the compound parameter approach [101]. In this assessment, a lower value indicates a higher priority. As depicted in Table 6, the prioritization order of critical subwatersheds in the Urpash area is as follows: WS_3_ > WS_4_ > WS_5_ > WS_6_ > WS_2_ > WS_1_, as illustrated in Fig 7b. Furthermore, the results of soil erosion assessment (Fig 7c) within the Urpash watershed utilizing the RUSLE model revealed a priority order of WS_6 _> WS_3 _> WS_2 _> WS_5 _> WS_4 _> WS_1_ (Fig 7d). This ranking reflects the varying degrees of soil loss across different subbasins of the watershed, providing valuable insights for targeted management and conservation strategies.

**Table 5:** Morphometric parameters of the subwatersheds within the Urpash watershed, providing detailed data on each subwatershed’s linear, areal, and relief parameters.

S. No.	Property	WS_1_	WS_2_	WS_3_	WS_4_	WS_5_	WS_6_
1.	Area (A) (km^2^)	5.06	2.76	5.18	4.03	2.96	1.38
2.	Basin length (km)	4.50	3.55	4.54	3.80	2.92	3.02
3.	Perimeter(P) (km)	18.06	8.52	11.36	12.56	7.82	6.68
4.	Relative relief (H) (m)	417.87	1097.00	1504.99	1026.99	1286.00	1071.00
5.	Relief ratio	0.09	0.31	0.33	0.27	0.44	0.35
6.	Stream length (km)	8.52	4.03	8.22	9.51	2.98	2.33
7.	Stream frequency	1.78	1.81	1.93	1.74	1.01	2.17
8.	No. of streams	9	5	10	7	3	3
9.	Shape factor	4.00	4.56	3.98	3.58	2.80	6.60
10.	Drainage density (km/km^2^)	1.68	1.46	1.59	2.36	1.09	1.69
11.	Drainage texture	0.49	0.58	0.88	0.56	0.38	0.45
12.	Circularity ratio	0.19	0.47	0.50	0.32	0.60	0.39
13	Elongation ratio	0.56	0.53	0.56	0.59	0.66	0.44
14.	Mean bifurcation ratio	1	1.5	1.58	2.25	2	2
15.	Form factor	0.25	0.22	0.25	0.28	0.35	0.15
16.	Ruggedness No.	0.70	1.60	2.38	2.40	1.30	1.80
17.	Infiltration No.	2.99	2.65	3.06	4.10	1.02	3.67
18.	Length of overland flow (km)	0.29	0.34	0.31	0.21	0.49	0.29

**Table 6:** Prioritization of subwatersheds in the Urpash watershed based on the compound parameter approach and soil loss, ranking each subwatershed in terms of its susceptibility to soil erosion.

S. No.	Name of watershed	Ranking of watersheds based on parameters							Average rating	Priority order	Soil Loss (t/ha/yr)	Priority order
		R_b_	D_d_	F_s_	D_t_	R_f_	R_c_	R_e_			[36]	
1.	WS_1_ (Malpora)	5	3	4	4	3	6	3	4	**6**	5.98	**6**
2.	WS_2_ (Shahpora)	4	5	3	2	4	3	4	3.57	**5**	36.92	**3**
3.	WS_3_ (Sadrabagh)	3	4	2	1	3	2	3	2.57	**1**	41.96	**2**
4.	WS_4_ (Nuner)	1	1	5	3	2	5	2	2.71	**2**	9.55	**5**
5.	WS_5_ (Badhrakund)	2	6	6	6	1	1	1	3.28	**3**	10.22	**4**
6.	WS_6_ (Upper Urpash)	2	2	1	5	5	4	5	3.43	**4**	45.35	**1**

A comparison between this priority order and the one obtained from morphometric analysis reveals discrepancies. The difference in orders arises from the exclusion of critical parameters like Rainfall, Runoff, and soil properties in morphometry-based prioritization. Hence, prioritization based solely on morphometry should not supersede priority based on soil loss. However, a slight similarity in trends between the two prioritization orders is worth noting. Therefore, prioritizing critical subwatersheds based on morphometric analysis of a basin offers an effective solution for assessing vulnerable areas, particularly in situations where physical sampling is challenging or expensive, or in hydrologically similar watersheds. By employing both methodologies, this study establishes a validated framework that enhances the reliability of subwatershed prioritization. The findings provide valuable insights for targeted management and conservation strategies, which are crucial given the increasing environmental pressures in the Urpash watershed. This integrated approach can be adapted for other ecologically sensitive and understudied regions, offering a scalable and transferable method for watershed management. These may include the construction of loose rock-filled dams strategically placed along 1^st^-order streams with lengths exceeding 100 meters, earthen embankment bunds designed for 2nd-order streams, and small concrete masonry dams suitable for 3^rd^-order streams with adequate runoff [36]. These structures aim to mitigate soil erosion and manage water resources effectively within the watershed [102]. By adopting such measures, policymakers and land managers can enhance the sustainability of land use practices and safeguard the ecological integrity of the Urpash watershed and similar hydrological regions.

## Conclusion

This study provides a comprehensive morphometric analysis of the Urpash watershed, an ungauged and ecologically sensitive area, offering crucial insights into its hydrological behaviour, landform characteristics, and erosion susceptibility.. Using GIS and remote sensing, key morphometric parameters were analyzed, revealing that sub-watersheds with higher linear and relief parameters are more prone to erosion. The integration of the compound parameter method and RUSLE model enabled the prioritization of erosion-prone areas, guiding targeted conservation efforts. While the prioritization order derived from morphometric analysis may differ from soil erosion assessment, both approaches offer valuable insights for targeted management. Furthermore, this research fills a significant gap, particularly as it addresses the under-researched Urpash watershed. Despite its contributions, the study is constrained by the use of a 30m resolution DEM, and the absence of field-based soil validation. Future research should incorporate higher-resolution datasets and AI/ML techniques for improved watershed assessment The outcomes of this study extend beyond the Urpash watershed, offering a methodology applicable to other watersheds with similar topographic and hydrological characteristics. The insights gained here are especially relevant in regions facing land degradation and water scarcity, providing a data-driven approach for optimizing conservation measures. Policymakers and conservationists can use these findings to implement focused, cost-effective interventions, thereby enhancing the resilience of watersheds to both human-induced and climate-related challenges. In conclusion, this study underscores the importance of morphometric analysis as a foundational tool for watershed prioritization and management, paving the way for informed decision-making in land and water conservation practices.

## References

[1] Javed A, Khanday MY, Ahmed R. Prioritization of sub-watersheds based on morphometric and land use analysis using remote sensing and GIS techniques. J Indian Soc Remote Sens. 2009;37(2):261–74. doi: 10.1007/s12524-009-0016-8

[2] Ebrahimi Gatgash Z, Sadeghi SH. Prioritization-based management of the watershed using health assessment analysis at sub-watershed scale. Environ Dev Sustain. 2022;25(9):9673–702. doi: 10.1007/s10668-022-02455-8

[3] Memon AV, Patel YS, Parangi T. Exploring watershed dynamics: a comprehensive review on morphometric analysis and geospatial techniques. ISH Journal of Hydraulic Engineering. 2024;30(5):744–57. doi: 10.1080/09715010.2024.2383184

[4] Clarke JI. Morphometry from maps. In: Dury GH, editor. Essays in Geomorphology. Elsevier; 1996. p. 235–74.

[5] Nag S. Morphometric analysis using remote sensing techniques in the chaka sub-basin, purulia district, West Bengal. J Indian Soc Remote Sens. 1998;26(1–2):69–76. doi: 10.1007/bf03007341

[6] Soni S. Assessment of morphometric characteristics of Chakrar watershed in Madhya Pradesh India using geospatial technique. Appl Water Sci. 2016;7(5):2089–102. doi: 10.1007/s13201-016-0395-2

[7] Agarwal CS. Study of drainage pattern through aerial data in Naugarh area of Varanasi district, U.P. J Indian Soc Remote Sens. 1998;26(4):169–75. doi: 10.1007/bf02990795

[8] Altaf F, Meraj G, Romshoo SA. Morphometric Analysis to Infer Hydrological Behaviour of Lidder Watershed, Western Himalaya, India. Geography Journal. 2013;2013:1–14. doi: 10.1155/2013/178021

[9] Raja Shekar P, Mathew A. Morphometric analysis of watersheds: A comprehensive review of data sources, quality, and geospatial techniques. Watershed Ecology and the Environment. 2024;6:13–25. doi: 10.1016/j.wsee.2023.12.001

[10] Xu Y, Yu Q, Liu C, Li W, Quan L, Niu C, et al. Construction of a semi-distributed hydrological model considering the combination of saturation-excess and infiltration-excess runoff space under complex substratum. Journal of Hydrology: Regional Studies. 2024;51:101642. doi: 10.1016/j.ejrh.2023.101642

[11] Moore ID, Grayson RB, Ladson AR. Digital terrain modelling: A review of hydrological, geomorphological, and biological applications. Hydrological Processes. 1991;5(1):3–30. doi: 10.1002/hyp.3360050103

[12] Malik MI, Bhat MS, Kuchay NA. Watershed-based drainage morphometric analysis of Lidder catchment in Kashmir valley using geographical information system. Recent Res Sci Technol. 2011;3:118–26.

[13] Desta L, Carucci V, Wendem-Agenehu A, Abebe Y. Community-based participatory watershed development: A guideline. Ministry of Agriculture and Rural Development; 2005.

[14] Abdeta GC, Tesemma AB, Tura AL, Atlabachew GH. Morphometric analysis for prioritizing sub-watersheds and management planning and practices in Gidabo Basin, Southern Rift Valley of Ethiopia. Appl Water Sci. 2020;10(7). doi: 10.1007/s13201-020-01239-7

[15] Meshram SG, Sharma SK. Prioritization of watershed through morphometric parameters: a PCA-based approach. Appl Water Sci. 2015;7(3):1505–19. doi: 10.1007/s13201-015-0332-9

[16] Rekha BV, George AV, Rita MJER. Morphometric analysis and micro-watershed prioritization of Peruvanthanam sub-watershed, the Manimala River Basin, Kerala, South India. Environ Res Eng Manag. 2011;57:6–14.

[17] Singh S, Singh MC. Morphometric analysis of Kanhar river basin. Natl Geogr J India. 1997;43:31–43.

[18] Horton RE. Erosional development of streams and their drainage basins; hydrophysical approach to quantitative morphology. Geol Soc America Bull. 1945;56(3):275. doi: 10.1130/0016-7606(1945)56[275:edosat]2.0.co;2

[19] Smith KG. Standards for grading texture of erosional topography. American Journal of Science. 1950;248(9):655–68. doi: 10.2475/ajs.248.9.655

[20] Prabhakaran A, Jawahar Raj N. Drainage morphometric analysis for assessing form and processes of the watersheds of Pachamalai hills and its adjoinings, Central Tamil Nadu, India. Appl Water Sci. 2018;8(1). doi: 10.1007/s13201-018-0646-5

[21] Rai PK, Chaubey PK, Mohan K, Singh P. Geoinformatics for assessing the inferences of quantitative drainage morphometry of the Narmada Basin in India. Appl Geomat. 2017;9(3):167–89. doi: 10.1007/s12518-017-0191-1

[22] Rao NK, Latha SP, Kumar AP, Krishna HM. Morphometric analysis of Gostani river basin in Andhra Pradesh State, India using spatial information technology. Int J Geomatics Geosci. 2010;1:179. doi: 10.36106/ijsr/9320212

[23] Singh MC, Satpute S, Prasad V. Remote sensing and GIS-based watershed prioritization for land and water conservation planning and management. Water Sci Technol. 2023;88(1):233–65. doi: 10.2166/wst.2023.207 37452545

[24] Jensen JR. Remote sensing of the environment: An earth resource perspective 2/e. Pearson Education India; 2009.

[25] Chowdary VM, Chakraborthy D, Jeyaram A, Murthy YVNK, Sharma JR, Dadhwal VK. Multi-Criteria Decision Making Approach for Watershed Prioritization Using Analytic Hierarchy Process Technique and GIS. Water Resour Manage. 2013;27(10):3555–71. doi: 10.1007/s11269-013-0364-6

[26] Balasubramanian A, Duraisamy K, Thirumalaisamy S, Krishnaraj S, Yatheendradasan RK. Prioritization of subwatersheds based on quantitative morphometric analysis in lower Bhavani basin, Tamil Nadu, India using DEM and GIS techniques. Arab J Geosci. 2017;10(24). doi: 10.1007/s12517-017-3312-6

[27] Nwilo PC, Ogbeta CO, Daramola OE, Okolie CJ, Orji MJ. Soil Erosion Susceptibility Mapping of Imo River Basin Using Modified Geomorphometric Prioritisation Method. Quaestiones Geographicae. 2021;40(3):143–62. doi: 10.2478/quageo-2021-0029

[28] Nasir MJ, Ahmad W, Jun C, Iqbal J, Bateni SM. Soil erosion susceptibility assessment of Swat River sub-watersheds using the morphometry-based compound factor approach and GIS. Environ Earth Sci. 2023;82(12). doi: 10.1007/s12665-023-10982-4

[29] Inyele J, Murimi S, Kweyu R. Identification of critical sub-watersheds prone to soil erosion using remote sensing data and geospatial techniques in Thiririka watershed, Kenya. IOSR J Appl Geol Geophys. 2023;11:13–23. doi: 10.9790/0990-1104011323

[30] Shekar PR, Mathew A, P. S. A, Gopi VP. Sub-watershed prioritization using morphometric analysis, principal component analysis, hypsometric analysis, land use/land cover analysis, and machine learning approaches in the Peddavagu River Basin, India. Journal of Water and Climate Change. 2023;14(7):2055–84. doi: 10.2166/wcc.2023.221

[31] Topno AR, Job M, Rusia DK, Kumar V, Bharti B, Singh SD. Prioritization and identification of vulnerable sub-watersheds using morphometric analysis and an integrated AHP-VIKOR method. Water Supply. 2022;22(11):8050–64. doi: 10.2166/ws.2022.303

[32] Worachairungreung M, Kulpanich N, Thanakunwutthirot K, Hemwan P. Monitoring Agricultural Land Loss by Analyzing Changes in Land Use and Land Cover. Emerg Sci J. 2024;8(2):687–99. doi: 10.28991/esj-2024-08-02-020

[33] Hassan WH, Khazaal ST, Al-Shammari MH. Effect of Climate Change on Wetland Areas in West Iraq Using Satellite Data and GIS Techniques. Civ Eng J. 2024;10(9):2966–78. doi: 10.28991/cej-2024-010-09-013

[34] Rattanarat J, Jaroensutasinee K, Jaroensutasinee M, Sparrow EB. Government Policy Influence on Land Use and Land Cover Changes: A 30-Year Analysis. Emerg Sci J. 2024;8(5):1783–97. doi: 10.28991/esj-2024-08-05-06

[35] Renard KG, Foster GR, Weesies GA, Porter JP. RUSLE: Revised universal soil loss equation. J Soil Water Conserv. 1991;46:30–3.

[36] Attar MI, Pandey Y, Naseer S, Bangroo SA. Soil erosion modelling using GIS-integrated RUSLE of Urpash watershed in Lesser Himalayas. Arab J Geosci. 2024;17(3). doi: 10.1007/s12517-024-11893-9

[37] Farooq M. Soils of Jammu & Kashmir. ENVIS Newsletter. 2016:1–8. doi: 10.13140/RG.2.1.2421.7362

[38] Osei JD, Anyemedu FOK, Osei DK. Integrating 2D hydrodynamic, SWAT, GIS and satellite remote sensing models in open channel design to control flooding within road service areas in the Odaw river basin of Accra, Ghana. Model Earth Syst Environ. 2023;9(4):4183–221. doi: 10.1007/s40808-023-01742-1

[39] Vilà-Cabrera A, Astigarraga J, Jump AS, Zavala MA, Seijo F, Sperlich D, Ruiz-Benito P. Anthropogenic land-use legacies underpin climate change-related risks to forest ecosystems. Trends Plant Sci. 2023;28(10):1132–43. doi: 10.1016/j.tplants.2023.05.005 37263916

[40] Dhanush SK, Murthy MM, Sathish A. Quantitative Morphometric Analysis and Prioritization of Sub-Watersheds for Soil Erosion Susceptibility: A Comparison between Fuzzy Analytical Hierarchy Process and Compound Parameter Analysis Method. Water Resour Manage. 2024;38(4):1587–606. doi: 10.1007/s11269-024-03741-y

[41] Nooka Ratnam K, Srivastava YK, Venkateswara Rao V, Amminedu E, Murthy KSR. Check dam positioning by prioritization of micro-watersheds using SYI model and morphometric analysis — Remote sensing and GIS perspective. J Indian Soc Remote Sens. 2005;33(1):25–38. doi: 10.1007/bf02989988

[42] Sharma SK, Gajbhiye S, Tignath S, Patil R. Hypsometric analysis for assessing erosion status of watershed using geographical information system. Hydrol Model. 2018;81:263–276. doi: 10.1007/978-981-10-5801-1_19

[43] Singh P, Gupta A, Singh M. Hydrological inferences from watershed analysis for water resource management using remote sensing and GIS techniques. The Egyptian Journal of Remote Sensing and Space Science. 2014;17(2):111–21. doi: 10.1016/j.ejrs.2014.09.003

[44] Dzwairo R, Singh SK, Patel A. Soil erosion susceptibility assessment through morphometric analysis and morphotectonic implications in Rietspruit sub-basin, South Africa. Environ Dev Sustain. 2024;27(7):16503–24. doi: 10.1007/s10668-024-04650-1

[45] Bahiru TK, Aldosary AS, Kafy A-A, Rahman MT, Nath H, Kalaivani S, et al. Geospatial approach in modeling linear, areal, and relief morphometric interactions in Dabus river basin ecology for sustainable water resource management. Groundwater for Sustainable Development. 2024;24:101067. doi: 10.1016/j.gsd.2023.101067

[46] Sujatha ER, Selvakumar R, Rajasimman UAB, Victor RG. Morphometric analysis of sub-watershed in parts of Western Ghats, South India using ASTER DEM. Geomatics, Natural Hazards and Risk. 2013;6(4):326–41. doi: 10.1080/19475705.2013.845114

[47] Raja A, Gunasekaran P, Ilanthirayan A. Morphometric analysis of Kallarpatti sub-watershed, Mathur Taluk, Krishnagiri District. Int J Dev Res. 2017;7:17158–64.

[48] AL-Juaidi AEM. Prioritization of sub-watershed in Eastern Jeddah using PCA-WSA hybrid modeling approach. Environ Dev Sustain. 2024. doi: 10.1007/s10668-024-05042-1

[49] Raja Shekar P, Mathew A. Morphometric analysis of watersheds: A comprehensive review of data sources, quality, and geospatial techniques. Watershed Ecology and the Environment. 2024;6:13–25. doi: 10.1016/j.wsee.2023.12.001

[50] Strahler AN. Dynamic basis of geomorphology. Geol Soc America Bull. 1952;63(9):923. doi: 10.1130/0016-7606(1952)63[923:dbog]2.0.co;2

[51] Strahler AN. Part II: Quantitative geomorphology of drainage basins and channel networks. Handbook of Applied Hydrology. McGraw-Hill; 1964.

[52] Schumm SA. Evolution of drainage systems and slopes in badlands at perth amboy, New Jersey. Geol Soc America Bull. 1956;67(5):597. doi: 10.1130/0016-7606(1956)67[597:eodsas]2.0.co;2

[53] Horton RE. Drainage-basin characteristics. Eos, Trans Am Geophys Union. 1932;13:350–61. doi: 10.1029/tr013i001p00350

[54] Faniran A. The index of drainage intensity: a provisional new drainage factor. Aust J Sci. 1968;31:326–30.

[55] Melton MA. An Analysis of the Relations Among Elements of Climate, Surface Properties and Geomorphology. Department of Geology, Columbia University; 1957. Technical Report 11, Project NR 389-042. doi: 10.7916/d8-0rmg-j112

[56] Patton PC, Baker VR. Morphometry and floods in small drainage basins subject to diverse hydrogeomorphic controls. Water Resources Research. 1976;12(5):941–52. doi: 10.1029/wr012i005p00941

[57] Leopold LB, Wolman MG, Miller JP. Fluvial Processes in Geomorphology. W. H. Freeman and Co.; 1964.

[58] Yadav SK, Singh SK, Gupta M, Srivastava PK. Morphometric analysis of Upper Tons basin from Northern Foreland of Peninsular India using CARTOSAT satellite and GIS. Geocarto International. 2014;29(8):895–914. doi: 10.1080/10106049.2013.868043

[59] Strahler AN. Quantitative geomorphology of erosional landscapes. In: 19th International Geological Congress, Section XIII. 1954. 341–54.

[60] Christopher O, Idowu AO, Olugbenga AS. Hydrological analysis of Onitsha North East drainage basin using geoinformatic techniques. World Appl Sci J. 2010;11(10):1297–302.

[61] Krishnan A, Arjun S. Morphometric analysis in the sub basins of the Kali River using Geographic Information System, Karnataka, India. Geomatica. 2024;76(2):100013. doi: 10.1016/j.geomat.2024.100013

[62] Sitati A, Yegon MJ, Masese FO, Graf W. Ecological importance of low-order streams to macroinvertebrate community composition in Afromontane headwater streams. Environmental and Sustainability Indicators. 2024;21:100330. doi: 10.1016/j.indic.2023.100330

[63] Alemayehu A, Tesfaye A. The morphometric investigation of the Gelda watershed in the Lake Tana sub basin: implications for managing soil and water resources. Model Earth Syst Environ. 2024;10(3):4207–22. doi: 10.1007/s40808-024-02012-4

[64] Saha S, Das J, Mandal T. Investigation of the watershed hydro-morphologic characteristics through the morphometric analysis: A study on Rayeng basin in Darjeeling Himalaya. Environmental Challenges. 2022;7:100463. doi: 10.1016/j.envc.2022.100463

[65] Sajadi P, Singh A, Mukherjee S, Sang Y-F, Chapi K, Salari M. Drainage network extraction and morphometric analysis in an Iranian basin using integrating factor analysis and geospatial techniques. Geocarto International. 2020;37(3):896–925. doi: 10.1080/10106049.2020.1750060

[66] Kabite G, Gessesse B. Hydro-geomorphological characterization of Dhidhessa River Basin, Ethiopia. International Soil and Water Conservation Research. 2018;6(2):175–83. doi: 10.1016/j.iswcr.2018.02.003

[67] Torrefranca I, Otadoy RE. GIS-based watershed characterization and morphometric analysis in Bohol Watersheds, Philippines. Geology, Ecology, and Landscapes. 2022;8(4):527–38. doi: 10.1080/24749508.2022.2158554

[68] Mahala A. The significance of morphometric analysis to understand the hydrological and morphological characteristics in two different morpho-climatic settings. Appl Water Sci. 2019;10(1). doi: 10.1007/s13201-019-1118-2

[69] Suji VR, Karuppasamy S, Sheeja RV. Prioritization using morphometric analysis and land use/land cover parameters for Vazhichal watershed using remote sensing and GIS techniques. Int J Innov Res Sci Technol. 2015;2:61–8.

[70] Chow VT. Handbook of Hydrology. McGraw-Hill; 1964.

[71] Roy SK, Chowdhury MdA. Morphometric analysis and watershed delineation of the Karnaphuli river basin: A comparative study using different DEMs in Chittagong, Bangladesh. River. 2024;3(4):426–37. doi: 10.1002/rvr2.109

[72] Gregory KJ, Walling DE. Drainage Basin Form and Process—A Geomorphological Approach. Edward Arnold Pub Ltd.; 1973. doi: 10.1126/science.184.4140.977.b

[73] Anusree KK, Ajayakumar A, Reghunath R, Santhosh V. Morphometric and morphotectonic characteristics of a Tropical River Basin, North Kerala, India using geospatial technology. International Journal of River Basin Management. 2024;:1–20. doi: 10.1080/15715124.2024.2400690

[74] Leopold LB, Maddock T. The hydraulic geometry of stream channels and some physiographic implications. USGS Prof Pap. 1953;252:57. doi: 10.3133/pp252

[75] Ganie PA, Posti R, Pandey PK. Exploring and modelling the hydro-morphological landscape of a Himalayan basin: a geospatial study of Nandakini Basin in Uttarakhand, India. Discov Geosci. 2024;2(1). doi: 10.1007/s44288-024-00032-2

[76] Langbein WB. Topographic characteristics of drainage basins. US Geological Society Water Supply Paper. 1947:968-C. Available from: http://pubs.usgs.gov/wsp/0968c/report.pdf

[77] Singh VP, Yadav S, Yadava RN. Hydrologic Modeling: Select Proceedings of ICWEES-2016. Springer; 2018. doi: 10.1007/978-981-10-5801-1

[78] Fenta AA, Yasuda H, Shimizu K, Haregeweyn N, Woldearegay K. Quantitative analysis and implications of drainage morphometry of the Agula watershed in the semi-arid northern Ethiopia. Appl Water Sci. 2017;7(7):3825–40. doi: 10.1007/s13201-017-0534-4

[79] Miller VC. A quantitative geomorphic study of drainage basin characteristics in the Clinch Mountain area, Virginia and Tennessee. New York: Columbia Univ; 1953; 3. doi: 10.1086/626413

[80] Farhan Y, Anaba O. A Remote Sensing and GIS Approach for Prioritization of Wadi Shueib Mini-Watersheds (Central Jordan) Based on Morphometric and Soil Erosion Susceptibility Analysis. JGIS. 2016;08(01):1–19. doi: 10.4236/jgis.2016.81001

[81] Nookaratnam K, Srivastava YK, Venkateswararao V, Amminedu E, Murthy BV. Use of remote sensing and GIS for watershed prioritization in the context of soil conservation. Water Resour Manag. 2007;21:1557–75. doi: 10.1007/s11269-006-9079-4

[82] Bogale A. Morphometric analysis of a drainage basin using geographical information system in Gilgel Abay watershed, Lake Tana Basin, upper Blue Nile Basin, Ethiopia. Appl Water Sci. 2021;11(7). doi: 10.1007/s13201-021-01447-9

[83] Patel DP, Dholakia MB, Naresh N, Srivastava PK. Water Harvesting Structure Positioning by Using Geo-Visualization Concept and Prioritization of Mini-Watersheds Through Morphometric Analysis in the Lower Tapi Basin. J Indian Soc Remote Sens. 2011;40(2):299–312. doi: 10.1007/s12524-011-0147-6

[84] Attar MI, Naseer S, Khan JN, Bangroo SA, Altaf Y, Khan AH, et al. Assessment of shift in GWPZs in Kashmir Valley of Northwestern Himalayas. Environmental and Sustainability Indicators. 2024;24:100513. doi: 10.1016/j.indic.2024.100513

[85] Chorley RJ. Introduction to physical hydrology. Methuen and Co. Ltd.; 1969.

[86] Ozdemir H, Bird D. Evaluation of morphometric parameters of drainage networks derived from topographic maps and DEM in point of floods. Environ Geol. 2008;56(7):1405–15. doi: 10.1007/s00254-008-1235-y

[87] Swetha K, Hemalatha K, Srinivasa Rao M, Bhagat RS, Padmini Y, Raja Rao G. Integrating Remote Sensing and GIS for Morphometric Analysis of Gadi Gedda Watershed, Vizianagaram District, Andhra Pradesh, India: A Comprehensive Approach. InModern River Science for Watershed Management: GIS and Hydrogeological Application. 2024:89–110. 10.1007/978-3-031-54704-1_7

[88] Zavoiance I. Morphometry of Drainage Basins. Developments in Water Science. Elsevier Sci; 1985.

[89] Bilewu S, Sule B, Ayanshola A. Optimum parameter selection for the morphometric description of watersheds: a case study of Central Nigeria. J Ecol Eng. 2015;16:29–35. doi: 10.12911/22998993/59344

[90] Schmid BH. Critical rainfall duration for overland flow from an infiltrating plane surface. Journal of Hydrology. 1997;193(1–4):45–60. doi: 10.1016/s0022-1694(96)03152-6

[91] Chitra C, Alaguraja P, Ganeshkumari K, Yuvaraj D, Manivel M. Watershed characteristics of Kundah subbasin using remote sensing and GIS techniques. Int J Geomatics Geosci. 2011;2:311–35. doi: 10.12691/aees-10-8-4

[92] Rai PK, Chandel RS, Mishra VN, Singh P. Hydrological inferences through morphometric analysis of lower Kosi river basin of India for water resource management based on remote sensing data. Appl Water Sci. 2018;8(1). doi: 10.1007/s13201-018-0660-7

[93] Boitt M, Bebeto N. Morphometric and Change Detection Analysis for Prioritization of Sub Basin Conservation, Case Study of Taita Hills. IJG. 2020;11(10):591–612. doi: 10.4236/ijg.2020.1110031

[94] Schumm SA, Hadley RF. Progress in the application of landform analysis in studies of semiarid erosion. United States Department of the Interior, Geological Survey; 1961. Cir 437. Available from: 10.3133/cir437

[95] Diaconu C. Altitude, one of the basic criteria for organizing hydrometeorological networks in mountainous regions. Studii de Hidrologie. 1966;18:41–55.

[96] Gottschalk LC. Reservoir sedimentation. Handbook of Applied Hydrology. McGraw-Hill Book Co.; 1964.

[97] Sarkar S, Gundekar HG. Geomorphological parameters: are they indicators for installation of a hydropower site? In: Proc International Conference on Small Hydropower—Hydro Sri Lanka. 2007:22–24.

[98] Mandale V, Bansod R. Quantitative Morphometric Analysis of the Adula Watershed, in Ahmednagar Maharashtra Using the ESRI- ArcGIS Tool. CJAST. 2019:1–10. doi: 10.9734/cjast/2019/v36i530252

[99] Strahler AN. Quantitative analysis of watershed geomorphology. Eos, Trans Am Geophys Union. 1957;38:913–20. doi: 10.1029/tr038i006p00913

[100] Rodrigues VS, do Valle Júnior RF, Sanches Fernandes LF, Pacheco FAL. The assessment of water erosion using Partial Least Squares-Path Modeling: A study in a legally protected area with environmental land use conflicts. Sci Total Environ. 2019;691:1225–41. doi: 10.1016/j.scitotenv.2019.07.216 31466203

[101] Chandniha SK, Kansal ML. Prioritization of sub-watersheds based on morphometric analysis using geospatial technique in Piperiya watershed, India. Appl Water Sci. 2014;7(1):329–38. doi: 10.1007/s13201-014-0248-9

[102] Chen D, Wei W, Chen L. Effects of terracing practices on water erosion control in China: A meta-analysis. Earth-Science Reviews. 2017;173:109–21. doi: 10.1016/j.earscirev.2017.08.007

